# Corneal targeted fenticonazole nitrate-loaded novasomes for the management of ocular candidiasis: Preparation, *in vitro* characterization, *ex vivo* and *in vivo* assessments

**DOI:** 10.1080/10717544.2022.2103600

**Published:** 2022-07-26

**Authors:** Sadek Ahmed, Maha M. Amin, Sarah Mohamed El-Korany, Sinar Sayed

**Affiliations:** a Department of Pharmaceutics and Industrial Pharmacy, Faculty of Pharmacy, Cairo University, Cairo, Egypt; b Department of Microbiology and Immunology, Faculty of Pharmacy, Cairo University, Cairo, Egypt

**Keywords:** Fenticonazole Nitrate, central composite design, gamma sterilization, corneal permeation, susceptibility test

## Abstract

The purpose of this manuscript was to develop and optimize Fenticonazole Nitrate (FTN)-loaded novasomes aiming to enhance drug corneal penetration and to improve its antifungal activity. Ethanol injection was used to formulate FTN-loaded novasomes adopting a central composite design. The researched factors were: stearic acid concentration (g%) (A), span 80: drug ratio (B) and cholesterol amount (mg) (C), and their effects on percent entrapment efficiency (EE%), particle size (PS), poly-dispersity index (PDI), zeta potential (ZP), and *in vitro* drug release after 8 hours (Q8h) were studied. Numerical optimization by Design-Expert® software was employed to select the optimum formula in respect to highest EE%, ZP (as absolute value), and Q8h >80% and lowest PS and PDI. Additional evaluation of the optimum formula was accomplished by short term stability study, effect of gamma sterilization, determination of Minimal Inhibitory Concentration and *ex vivo* corneal permeation study. The *in vivo* evaluation of the optimum formula was done to ensure its safety via *in vivo* ocular irritancy and *in vivo* corneal tolerance studies. Also, the efficacy was confirmed through *in vivo* corneal uptake study and susceptibility test. The optimum formula with the highest desirability value (0.738) showed EE% (94.31%), PS (197.05 nm), ZP (-66.95 mV) and Q8h (85.33%). It revealed to be safe, with augmented corneal permeation (527.98 µg/cm^2^) that leads to higher antifungal activity. The above results confirmed the validity of novasomes to improve the corneal permeation and antifungal activity of Fenticonazole Nitrate.

## 1. Introduction

The human’s eye is a very sensitive organ with a complex structure that can suffer from many ophthalmic diseases ranging from slight disorders like conjunctivitis to complaints that could cause blindness. Therefore, suitable medication should be selected and delivered to the distressed part (Albash et al., 2021). There are several structural and physiological obstacles throughout the eye that limit ocular absorption, such as stimulated tear formation, natural tear turnover and fast precorneal clearance. In addition, portion of the given dose could reach the systemic circulation by the nasolacrimal pathway, resulting in potential side effects (Elsayed & Sayed, 2017). Topical eye drops are frequently used because of their simple instillation and noninvasive administration. However, serious damage in the ocular surface could result from the frequent usage of concentrated solutions (Kaur et al., 2004). Vesicular Nano-carriers are believed to overwhelm the obstacles of ophthalmic delivery and to improve the ocular regulated delivery of ophthalmic medications.

The cornea is a physical obstacle made of three layers: epithelium, stroma and endothelium. The epithelium is formed of five to seven layers of firmly attached cells letting only the movement of small hydrophobic molecules (Prausnitz & Noonan, 1998). However, the stroma is a watery dense layer, while the endothelium is delicate layer of cells; so, both of them are not regarded as a critical barrier to permeation (Gaudana et al., 2010). Ocular absorption necessitates sustained precorneal residence that leads to effective corneal penetration (Elsayed & Sayed, 2017). Many researches were focused on improving systems with effective corneal permeation and prolonged precorneal residence time to enhance the ophthalmic bioavailability like mixed micelles (Younes et al., 2018), cubosomes (Emad Eldeeb et al., 2019; Sayed et al., 2021), solid lipid nanoparticles (Ahmad et al., 2019), and bilosomes (Abdelbary et al., 2016).

Novasomes represent a recent vesicular carrier that was first established by the IGI laboratories NOVAVAX to improve certain delivery systems (Mosallam et al., 2021). They have an improved liposomal or niosomal structure composed of cholesterol, free fatty acid (FFA), and monoester of polyoxyethylene fatty acid (Mosallam et al., 2021). Many vaccines have been synthesized as novasomes (Gregoriadis, 1995; Chambers et al., 2004). Stearic acid is a saturated free fatty acid (FAA) that is used widely in formulations due to its safety (Singh et al., 2020). Cholesterol is a chief component of the plasma membrane of mammalian cells. It’s involved in many biological processes like preserving fluidity of plasma membrane and biosynthesis of bile acids, steroid hormones, and vitamin D (Xu et al., 2022). It has a critical impact on vesicle stability, loading capacity and permeability (Abdelbary et al., 2017). Span 80 is a biodegradable nonionic surface active agent derived from oleic acid and sorbitol. Surfactants have a critical effect on the structure and the properties of many vesicular systems, they also serve as penetration enhancers (Abd-Elsalam & Ibrahim, 2021).

Fungal ophthalmic diseases are considered very dangerous since that could lead to blindess (Albash et al., 2021). The increased immunocompromised population from viral infections, chemotherapy, transplantation surgeries and anti-cancers resulted in a remarkable spread in the occurrence of fungal ophthalmic infections. Recently, anti-fungal drugs are consumed separately or in combination with antibiotics and corticosteroids (Veraldi & Milani, 2008). Fenticonazole nitrate (FTN) is an antifungal imidazole compound whose mechanism of action involves preventing ergosterol production, resulting in harming the cytoplasmic membrane of the fungi (Campos et al., 2018). FTN has both fungistatic and fungicidal actions on dermatophytes, yeasts and fungi, in addition to its activity against Gram positive bacteria. FTN has a low aqueous solubility that is less than 0.10 mg/mL, and this could have an undesirable consequence on its effectiveness, pharmacokinetic profiles and the development of resistance. Many techniques were formerly used to enhance its efficacy like terpesomes (Albash et al., 2021) and cerosomes (Albash et al., 2021). Terpesomes developed by Albash *et al*., showed small particle size, high EE %, improved corneal adhesion and greater *in vivo* retention compared to drug suspension (Albash et al., 2021). Cerosomes formulated by Albash *et al*., revealed minute particle size, acceptable zeta potential and improved safety (Albash et al., 2021).

Central composite design (CCD) is an example of response surface methodology that can be used for formulation optimization. Advantages of CCD include: require few design points, high accuracy and provide a rational amount of data for assessing the suitability of fit (Imanian & Biglari, 2022) compared to the counterpart three level full factorial design.

Specifically, the goal of this research is to develop and optimize FTN-loaded novasomes via central composite design, by studying the effect of stearic acid concentration (g%), span 80: drug ratio and cholesterol amount (mg) on percent entrapment efficiency (EE %), particle size (PS), poly-dispersity index (PDI), zeta potential (ZP), and Q8h *in vitro* drug release. The optimized formula was further evaluated *in vitro* regarding (DSC, FTIR, TEM), short term stability, MIC determination and effect of gamma sterilization. In addition*, ex vivo* corneal permeation study and *in vivo* evaluation of the optimum formula to ensure its safety via *in vivo* ocular irritancy and corneal tolerance studies were assessed. Finally, the efficacy of the optimum FTN-loaded formula was confirmed through *in vivo* corneal uptake study and susceptibility test.

## 2. Materials and methods

### 2.1. Materials

FTN was a gift from Andalous Pharmaceutical Co. (Cairo, Egypt). Span 80, cholesterol, methanol (HPLC grade), dialysis membranes (typical molecular weight cutoff 14,000 Da) and Rhodamine B were purchased from Sigma Chemical Company. Ethanol (95%), isopropyl alcohol, formaldehyde and stearic acid were provided from El-Nasr pharmaceutical chemicals Co. (Cairo, Egypt). All other chemicals and solvents were of analytical grade and were consumed as obtained.

### 2.2. Animals

Adult male albino rabbits, having an average body weight of 2 ± 0.5 kg, were housed individually (one per cage) at 25 ± 2 °C, with 12 hours cycle alternating of light and dark. Animals were supplied with the standard commercial food and tap water. Initial examination of all rabbits’ eyes was carried out. Rabbits with no signs of ocular inflammation were included in the study. This study was approved by Research Ethics Committee (REC), Faculty of Pharmacy, Cairo University (approval number PI 3132) and was conducted compliant with the Guide for Care and Use of Laboratory Animals published by the US National Institute of Health (NIH Publication No. 85–23, revised 2011).

### 2.3. Methods

#### 2.3.1. Experimental design

FTN-loaded novasomes were formulated adopting a central composite design. This design comprises twenty trails, which are: 8 factorial points, 6 axial points and 6 replicated center point. Alpha was set at 1.68179. The studied factors were: stearic acid concentration (%) (A), span 80: drug ratio (B) and cholesterol amount (mg) (C) all at three levels. These levels were selected according to preliminary studies results and were defined as (-1, 0, +1). The studied responses were percent entrapment efficiency (EE %) (Y1), particle size (PS) (Y2), poly-dispersity index (PDI) (Y3), zeta potential (ZP) (Y4), and *in-vitro* release after 8 hours (Q8h) (Y5). Design-Expert^®^ software version 7 (Stat-Ease, Inc., Minneap-olis, Minnesota, USA) was employed in order to detect the significance of the studied factors (Ahmed et al., 2020). Central composite design with levels of independent variables and their corresponding responses are presented in Table 1.

**Table 1.** Factorial levels of studied independent variables in the central composite design together with measured responses and their desirability constraints.

Factor (independent variable)	Level	
	-1	+1
A: stearic acid concentration (%)	0.125	0.375
B: span 80: drug ratio	3:1	9:1
C: cholesterol amount (mg)	20	60
**Response (dependent variable)**	**Desirability constraints**	
Y1: EE %	Maximize	
Y2: PS (nm)	Minimize	
Y3: PDI	Minimize	
Y4: ZP (absolute value) (mV)	Maximize	
Y5: Q8h (%)	>80%	

**Abbreviations:** EE %, percent entrapment efficiency; PDI, poly-dispersity index; PS, particle size; Q8h, percent drug released after 8 hours; ZP, zeta potential.

#### 2.3.2. Formulation of FTN-loaded novasomes

Novasomes of FTN were prepared utilizing ethanol injection method (Mosallam et al., 2021) with minor adjustment. In brief, FTN (10 mg), varying amount of stearic acid, span 80 and cholesterol were accurately weighted and dissolved in (10 mL) ethanol using water bath at 60 °C. Afterwards, the ethanolic solution was injected gradually into a four-fold greater volume of phosphate-buffered saline (PBS, pH 7.4) magnetically stirred at the same temperature until total vaporization of ethanol. The sudden turbidity indicated the formation of novasomes, the resulted novasomal dispersions were sonicated for 15 min at 25 ± 2 °C for size reduction and stored till further use at 4 °C (Al-Mahallawi et al., 2014). Table 2 presents the composition of the fabricated FTN-loaded novasomal formulae (T1–T20) listed in random order.

**Table 2.** Composition of the different prepared FTN-loaded novasomes with their measured responses of central composite design (*n* = 3 ± SD).

Trial	Factors				Responses			
	A: stearic acid concentration (%)	B: span 80: drug ratio	C: cholesterol amount (mg)	Y1: EE % (Mean +SD)	Y2: PS (nm) (Mean +SD)	Y3: PDI (Mean +SD)	Y4: ZP (mV)(Mean +SD)	Y5: Q8h (%)(Mean +SD)
T1	0.040	6:1	40	88.89 ± 1.18	205.45 ± 2.76	0.13 ± 0.00	−44.40 ± 4.17	92.48 ± 2.16
T2	0.125	9:1	60	96.53 ± 2.16	301.45 ± 15.06	0.40 ± 0.12	−64.20 ± 1.27	84.23 ± 2.25
T3	0.125	3:1	20	82.64 ± 2.36	160.40 ± 2.12	0.36 ± 0.06	−32.80 ± 1.56	95.10 ± 1.21
T4	0.125	3:1	60	93.96 ± 3.24	160.40 ± 3.39	0.22 ± 0.05	−63.85 ± 0.64	84.81 ± 2.79
T5	0.125	9:1	20	85.21 ± 1.28	299.00 ± 11.03	0.34 ± 0.01	−43.60 ± 0.14	94.31 ± 1.70
T6	0.250	6:1	40	89.58 ± 1.37	207.00 ± 0.14	0.27 ± 0.04	−48.75 ± 4.45	90.51 ± 1.35
T7	0.250	6:1	40	90.28 ± 2.55	212.65 ± 0.78	0.11 ± 0.01	−50.70 ± 2.12	89.21 ± 1.45
T8	0.250	6:1	40	90.49 ± 3.24	214.60 ± 0.85	0.14 ± 0.00	−52.80 ± 1.98	88.65 ± 0.74
T9	0.250	6:1	40	90.63 ± 0.69	215.35 ± 0.49	0.14 ± 0.00	−55.25 ± 2.19	88.10 ± 3.39
T10	0.250	6:1	73.64	97.92 ± 0.79	288.65 ± 5.87	0.42 ± 0.03	−83.30 ± 0.57	80.78 ± 2.83
T11	0.250	6:1	40	90.76 ± 1.47	238.05 ± 3.32	0.16 ± 0.00	−58.25 ± 2.19	87.68 ± 1.75
T12	0.250	0.95:1	40	88.06 ± 1.96	124.35 ± 0.92	0.11 ± 0.03	−46.10 ± 3.96	91.29 ± 1.61
T13	0.250	11.05:1	40	92.36 ± 2.55	338.95 ± 25.95	0.32 ± 0.01	−59.65 ± 0.92	86.54 ± 2.33
T14	0.250	6:1	6.36	78.47 ± 0.98	206.10 ± 2.69	0.41 ± 0.08	−29.45 ± 1.91	95.57 ± 2.48
T15	0.250	6:1	40	90.97 ± 0.79	286.15 ± 14.64	0.56 ± 0.02	−59.45 ± 0.64	87.30 ± 2.19
T16	0.375	9:1	60	97.08 ± 3.54	323.65 ± 4.60	0.06 ± 0.01	−74.50 ± 0.57	82.66 ± 1.61
T17	0.375	9:1	20	86.67 ± 1.77	314.80 ± 31.54	0.45 ± 0.45	−44.35 ± 4.17	92.92 ± 0.71
T18	0.375	3:1	20	84.72 ± 1.57	170.25 ± 2.90	0.25 ± 0.03	−44.15 ± 0.35	93.61 ± 3.05
T19	0.375	3:1	60	95.14 ± 2.55	194.80 ± 5.94	0.28 ± 0.02	−64.95 ± 2.47	83.90 ± 2.37
T20	0.460	6:1	40	91.74 ± 2.26	291.50 ± 2.83	0.07 ± 0.09	−62.25 ± 0.07	85.99 ± 0.81

**Abbreviations:** EE %, percent entrapment efficiency; PDI, poly-dispersity index; PS, particle size; Q8h, percent drug released after 8 hours; ZP, zeta potential.

#### 2.3.3. In vitro characterization of the prepared FTN-loaded novasomes

##### 2.3.3.1. Percent entrapment efficiency (EE %)

Percent entrapment efficiency (EE %) of FTN was calculated by indirect measurement of free FTN (unentrapped FTN) (Abdelbary & AbouGhaly, 2015). Briefly, 1 mL of resulted formula was exposed to centrifugation via a cooling centrifuge (3K30, Sigma, Germany) at 21,000 rpm for 1 hour at 4 °C. The clear supernatant was isolated and diluted. The concentration of unentrapped FTN was spectrophotometrically assessed (Shimadzu, model UV-1601 PC, Kyoto, Japan) at λmax 252 nm using the calibration curve (*n* = 3, R^2^= 0.9998). The EE % was calculated applying the following equation (Sayed et al., 2020):

(Eq. 1) $$\text{EE \%}=\frac{\left(\text{total amount of FTN}-\text{ total amount of free FTN}\right)}{\text{total amount of FTN}}\text{ X }100$$

Total amount of FTN is the real weighed quantity, total amount of free FTN (quantity of FTN in supernatant)

##### 2.3.3.2. Particle size (PS), poly-dispersity index (PDI) and Zeta-potential (ZP)

The resulted dispersions were diluted 10 times with distilled water in order to clarify their PS, PDI, and ZP. The determination was completed by light scattering based on laser diffraction employing Malvern Zetasizer (Model ZEN3600, Malvern Instruments Ltd. Worcestershire, UK) (Abd-Elsalam & ElKasabgy, 2019).

##### 2.3.3.3. In vitro release studies

*In vitro* release of FTN from the formulated FTN-loaded novasomal dispersions were determined using bag dialysis method (typical molecular weight cutoff 14,000 Da; Sigma-Aldrich Co.) (Elsayed & Sayed, 2017). Concisely, dialysis membrane was soaked overnight in the release medium (phosphate buffer saline solution (pH 7.4) containing 25% ethanol to maintain the sink condition) (Albash et al., 2021; Rathod et al., 2017). Then, a dialysis bag enclosing 2 mL (equivalent to 0.5 mg of FTN) of each formula or FTN suspension was placed in 25 mL release medium in amber bottles. After that, the bottles were placed in shaker operating at 37 ± 0.5 °C and 100 rpm. Aliquots of 3 mL were withdrawn at planned time intervals (0.5, 1, 2, 4, 6, 8 h) and replaced with similar volume of fresh release medium in an attempt to sustain sink condition. The percent released was calculated by spectrophotometric measurement at λmax 252 nm against the calibration curve (R^2^= 0.9992). All release profiles were fitted to zero, first, and Higuchi diffusion models. The largest coefficient (R^2^) suggests the appropriate model (Ahmed et al., 2021).

#### 2.3.4. Selection of the optimum formula

Design-Expert^®^ software version 7 (Stat-Ease, Inc., Minneap-olis, Minnesota, USA) was employed using numerical optimization to detect desirability regarding the significant factors and ignoring the non-significant ones. Selection was based in terms of highest EE % and ZP (as absolute value), *in vitro* release (Q8h) > 80% and lowest PS and PDI. The formula with uppermost desirability (closet to 1) was chosen for additional assessment (Emad Eldeeb et al., 2019).

#### 2.3.5. In vitro characterization of the optimum formula

##### 2.3.5.1. Differential scanning calorimetry (DSC)

At the beginning, the optimum formula was freezed at (-20 °C) followed by lyophilization at (-45 °C) under lowered pressure for (24 h) using freeze-dryer (Novalyphe-NL 500 freeze-dryer, Savant Instruments, NY, USA) (Sayed et al., 2018). A specified weight (2 mg) of FTN, stearic acid, cholesterol, lyophilized FTN-loaded optimum formula and lyophilized FTN-free optimum formula were heated to (350 °C) in a nitrogen environment in an aluminum pan at a scanning rate of (10 °C/min) in order to obtain their thermal characters. Thermograms were noted by DSC7 (Perkin-Elmer, Waltham, MA) (Younes et al., 2018).

##### 2.3.5.2. Fourier transform infrared spectroscopy (FTIR)

FTIR spectra of FTN, stearic acid, cholesterol, lyophilized FTN-loaded optimum formula and lyophilized FTN-free optimum formula were recorded using FTIR spectrophotometer (model 22, Bruker, Coventry, UK). About, 2–3 mg of each sample was blended with dry potassium bromide and squeezed into disk ahead of being examined in the range of 4000 − 500 cm^−1^ at 25 ± 2 °C (Younes et al., 2018).

##### 2.3.5.3. Transmission electron microscopy (TEM)

The optimum formula was imaged by TEM in order to detect its size and shape. Firstly, samples were diluted ten times with distilled water. Then, placed over carbon coated copper rods, dried at 25 ± 2 °C and then, stained by 2% w/v phosphotungstic acid. Images were Taken by TEM (JEOL, Tokyo, Japan) operated at an accelerating voltage of 100 kV (Sayed et al., 2018).

##### 2.3.5.4. pH measurement

pH determination is significant to guarantee both safety and efficacy of the optimum formula. Generally, acidic (pH < 4) or alkaline (pH > 10) solution would harm the eye (Said et al., 2021), while pH from 4 to 8 would considerably improve ocular permeation (Mohanty et al., 2013). Usually, pH of ocular preparations has a range from 3.50 to 8.50 (Said et al., 2021). The pH of the optimum formula was determined using a pH meter (model-3505, Jenway, Staffordshire, UK). The obtained results were the averages of triplicates ± SD (Fahmy et al., 2021).

##### 2.3.5.5. Effect of short-term storage

Short-term storage study is important to ensure the capability of ocular products to maintain their properties and activity after storage under certain conditions (Albash et al., 2021). The optimum formula was preserved at a storage temperature range of (4–8 °C) for three months. At the end of storage period, Reevaluation of the stored optimum dispersion formula was done regarding physical appearance, EE %, PS, ZP and Q8h compared to the freshly prepared formula (Fahmy et al., 2021; Al-Mahallawi et al., 2017). EE %, PS, ZP were compared utilizing one-way ANOVA analysis. However, the release profiles of the fresh and stored optimum formula were compared using the Similarity factor “ƒ_2_”. Similarity factor “ƒ_2_” was calculated by applying the following equation (Sayed et al., 2021; Sayed et al., 2020):

(Eq. 2) $$f_{2}=50.\log\{[1+(\frac{1}{n})\sum_{t=1}^{n}{\left(R_{t}-T_{t}\right)}^{2}{]}^{-0.5}.100$$

R_t_ and T_t_ are the % FTN released from the fresh and stored optimum formula correspondingly at time t. The release profiles are similar when “ƒ2” value lies between 50 and 100 (Abdelbary et al., 2016).

##### 2.3.5.6. Effect of gamma sterilization

The optimum formula was subjected to gamma sterilization in the presence of dry ice to avoid any unwanted effect due to the elevation of temperature after gamma irradiation. Irradiation was performed using Cobalt-60 irradiator at rate of 1.774 kGy/h. Radiation dose was 25 kGy in an Indian Gamma cell (Sayed et al., 2020). Following gamma sterilization, the optimum formula was reassessed for its appearance, EE %, PS and ZP. EE %, PS, ZP were compared utilizing one-way ANOVA analysis. The release profiles before and after gamma sterilization were compared using the previously mentioned Similarity factor “ƒ_2_” equation. R_t_ and T_t_ are the % FTN released from the optimum formula before and after gamma sterilization correspondingly at time t. The release profiles are similar when “ƒ2” value lies between 50 and 100 (Abdelbary et al., 2016).

##### 2.3.5.7. Minimum inhibitory concentration (MIC) determination

The measurement of MIC was conducted using “Broth Microdilution Technique” according to the Clinical and Laboratory Standards Institute guidelines (Humphries et al., 2018). A volume of 150 μL of two fold strength Sabouraud dextrose broth (SDB) was added to each well of a sterile U-shaped bottom 96-well plate. Also 150 μL of each of the tested formulae (FTN suspension and the optimum formula) was added to the first well of each row. Two-fold serial dilutions of each of the tested formulae were done from one row to the next one till reaching the tenth row (250–0.49 μg/mL). The wells were then inoculated with 10 μL of *Candida albicans* ATCC 60193 suspension (10^7^ CFU/mL). Each row included one well as a negative control for sterility (neither yeast nor tested formula was placed) and another well as a positive control for growth (inoculated with yeast suspension only). Plates were incubated at 25 ± 2 °C for 24 hours in aerobic environment. The lowest concentration showing no observable microbial growth was the MIC. The experiment was repeated in three independent times (Albash et al., 2021).

#### 2.3.6. Ex vivo characterization of the optimum FTN-loaded novasomes

##### 2.3.6.1. Ex vivo corneal permeation

The study procedure was approved by Research Ethics Committee, Faculty of Pharmacy, Cairo University (REC-FOPCU) with a number of PI 3132. Male albino rabbits (weight 2 ± 0.5 kg) were selected for the investigation after anesthetizing them by intramuscular injection of 35 mg/kg ketamine and 5 mg/kg xylazine (Elsayed & Sayed, 2017; Emad Eldeeb et al., 2019; Sayed et al., 2020). Afterwards, decapitation was done to isolate the cornea and sclera that were washed using PBS (pH 7.4) and directly attached to one end of the open ended tube. The receptor medium was composed of 15 mL of phosphate buffer saline solution (pH 7.4) containing 25% ethanol to maintain sink condition. The Donor medium (either the optimum formula or FTN suspension) containing an equivalent amount of 0.5 mg of FTN was used. Aliquots of 0.5 mL were withdrawn at intervals of (1, 2, 4, 6, 8, 10 h) and replaced rapidly with fresh receptor medium to preserve sink condition (Sayed et al., 2020). The gathered samples were clarified using 0.45 μm membrane filter at each time and examined by HPLC (Shimadzu, Tokyo, Japan) operated with RP-C18 column (250 × 4.6 mm, 5 μm) and UV detector at λmax (252 nm). The mobile phase consisted of methanol-water (85:15, v/v) at a flow rate of 1.2 mL/min. The injection volume was 20 μL. The mobile phase was filtered through 0.45 μm membrane filter then 10 min sonication before running (Silva, 2019). The amount of FTN permeated per unit area (µg/cm^2^) was plotted versus time (h). Cumulative amount of FTN permeated through the corneal membrane per unit area (Q_10h_-_permeation_), flux at 10 hours (J_max_) was calculated for both optimum formula and drug suspension. Enhancement ratio was calculated for the optimized formula compared to the drug suspension. All studies were done in triples. The flux (J_max_) and the enhancement ratio (ER) were assessed from the following equations (Abdelbary et al., 2016; Younes et al., 2018; Sayed et al., 2018): Jmax =

(Eq. 3) $$\frac{\mathrm{Amount}of\mathrm{drug}\mathrm{permeated}}{\mathrm{Time}X\mathrm{Area}}$$

ER =

(Eq. 4) $$\frac{\mathrm{Jmax}of\mathrm{formulation}}{\mathrm{Jmax}of\mathrm{drug}\mathrm{suspension}}$$

##### 2.3.6.2. Ex vivo corneal hydration level

Instantaneously after the ex vivo permeation study, each cornea was detached, washed to remove any surface-remained formula, softly wiped with a filter paper to eradicate excess water and then weighed to determine wet corneal weight (W_w_). Then, the cornea was dehydrated at 50 °C for 24 h and reweighed to determine dry corneal weight (W_d_). The corneal hydration levels (HL%) of the optimum formula and FTN suspension were compared using one-way ANOVA test. HL% was determined using the following equation (Moustafa et al., 2017):

(Eq. 5) $$HL\%=[1-(\frac{W_{d}}{W_{w}})].100$$

#### 2.3.7. In vivo characterization of the optimum FTN-loaded novasomes

##### 2.3.7.1. Ocular irritancy test

This test was achieved to validate the safety of the optimum formula. Any probable optical irritancy and/or destructive consequences of the optimum formula was estimated by detecting any redness, tenderness or boosted tear formation after application to the eyes of albino rabbits. The test was conducted on three albino rabbits. The experiment was achieved by a single instillation of the tested preparation (optimum FTN-loaded formula) into one eye, while the contralateral eye functioned as control. Both eyes of the rabbits under test was examined for any sign of irritation, such as conjunctival corneal edema and/or hyperhemia upon direct visual observation using a slit lamp, before treatment and at 1, 8 and 24 h following drug instillation (Abdelbary et al., 2017).

##### 2.3.7.2. In vivo corneal tolerance

A histopathological study was performed to notice any corneal tissue injury caused by the optimum formula. The test was conducted on three albino rabbits. In brief, the optimum formula was assessed by comparison to sterile normal saline (negative control) and isopropyl alcohol 95% (positive control). One drop from of normal saline or isopropyl alcohol 95% was applied into one eye of a male albino rabbit. While the optimum formula was applied into the other eye. Application of each liquid was done twice daily for one week (Elsayed & Sayed, 2017; Sayed et al., 2020). Animals were firstly anesthetized as mentioned in *ex vivo* study. Following sacrificing the animals by decapitation, corneas were removed from the detached eyes and cautiously washed using normal saline to avoid their damage. Corneal tissues were preserved in 10% v/v formalin saline solution till assessment. Solid paraffin cubes enclosing the corneas were obtained by immersing the corneas in molten paraffin, followed by cooling. Thin slices were obtained using a microtome. Eosin and hematoxylin were used as staining agents. A digital light microscope (Leica, Cambridge, UK) was used for the examination of the specimens (Sayed et al., 2021; Sayed et al., 2020).

##### 2.3.7.3. In vivo corneal uptake

The drug FTN in the optimum formula was substituted by Rhodamine B (RhB) in concentration of 0.1% w/w to be examined under Confocal laser scanning microscopy (CLSM) (LSM 710; Carl Zeiss, Jena, Germany). One drop of RhB-loaded formula (100 µL) was applied into the right eye male albino rabbit (2–3 kg), while the left eye received RhB-loaded aqueous solution (negative control). After 6 h, the animals were sacrificed by decapitation after being anesthetized. The clear corneas were carefully removed and cleaned. Corneal tissues were preserved in artificial tears and imaged on the same day. RhB was fluorescently visualized by excitation at 485 and 595 nm using argon and helium–neon lasers, respectively. Confocal images were processed and rectified using LSM software version 4.2 (Carl Zeiss Microimaging, Jena, Germany) (Elsayed & Sayed, 2017; Younes et al., 2018).

##### 2.3.7.4. Susceptibility test

Six rabbits were divided randomly into 2 groups (3 rabbits in each group, *n* = 3) where group I received the optimum formula and group II received FTN suspension. *Candida albicans* ATCC 60193 was used as the test organism. The experiment was performed as described by Basha et al (Basha et al., 2013) but with slight modifications. Briefly, fifty microliters of each of the tested formulae (the optimum formula and FTN suspension) were inserted within the lower conjunctival sac of the right eye of the rabbit using a micropipette. No drug was inserted in the left eye of each rabbit to serve as the control. At specific time intervals (1–10 hours), four sterile filter paper disks (Whatman no. 5, 6 mm in diameter) were wetted by placing the disks under the eyelid of the eye of each rabbit. For each eye (right and left), two disks were put in an Eppendorf tube (1.5 mL) which contains 500 μL Sabouraud dextrose broth (SDB) inoculated with 10% v/v yeast suspension (10^7^ CFU/mL). The other two disks were put in an Eppendorf tube containing 500 μL uninoculated SDB; this was used as a blank during measuring the optical densities. Afterwards, aerobic incubation of all the tubes was carried out at 25 ± 2 °C for 24 hours. After incubation, 200 mL of each tube was transferred to sterile 96-well plates and the optical densities (OD_600nm_) were measured using an automated spectrophotometric plate reader (Biotek, Synergy 2, USA) at a single wavelength of 600 nm. The obtained results were presented as average percentage growth inhibition (mean ± standard deviation).

The growth inhibition % was calculated using the following equation (Abdelbary et al., 2016; Fahmy et al., 2021):

(Eq. 6) $$\text{Growth inhibition \%}=\frac{\text{Control Left Eye }({\mathrm{OD}}_{600nm})-\text{ Test Right Eye }({\mathrm{OD}}_{600nm})}{\text{Control Left Eye }({\mathrm{OD}}_{600nm})}.100$$

The area under the curve from 1 to 10 h (AUC_(1–10h)_) was calculated from the curve of each individual animal using GraphPad Prism 7 software. Student’s *t*-test was used to compare between the optimum formula and FTN suspension

#### 2.3.8. Statistical analysis

Analysis of central composite design was performed using Design-Expert software version 7 (Stat- Ease Inc., Minneapolis, USA) (Ahmed et al., 2021). All measurements were done in triplicates. Results were represented as mean ± standard deviation (SD) and compared using ANOVA test (*p <* 0.05 indicated significance). One-way ANOVA was used for two independent groups comparisons were appropriate.

## 3. Results and discussions

### 3.1. Analysis of central composite design

The effects of stearic acid concentration (g%) (A), span 80: drug ratio (B) and cholesterol amount (mg) (C) as the independent variables on the different responses of the prepared FTN-loaded novasomes were determined using central composite design (Ahmed et al., 2021). Testing of each response was done exclusively and fitted to the model having highest prediction R^2^ (Zhang et al., 2010). The predicted R^2^ values were in reasonable synchronization with the adjusted R^2^ for all the studied responses as shown in Table 3.

**Table 3.** Model evaluations for measured responses.

Response	R^2^	Adjusted R^2^	Predicated R^2^	Adequate precision	Significant factors
**EE %**	0.9908	0.9883	0.9788	70.440	A, B, C, C^2^
**PS (nm)**	0.8947	0.8750	0.8547	22.650	A, B, C
**ZP (mV)**	0.9320	0.9192	0.8949	28.473	A, B, C
**Q8h (%)**	0.9223	0.9132	0.8986	32.395	A, C

**Abbreviations:** EE %, percent entrapment efficiency; PS, particle size; Q8h, percent drug released after 8 hours; ZP, zeta potential.

#### 3.1.1. Model analysis of EE %

**Figure 1.**
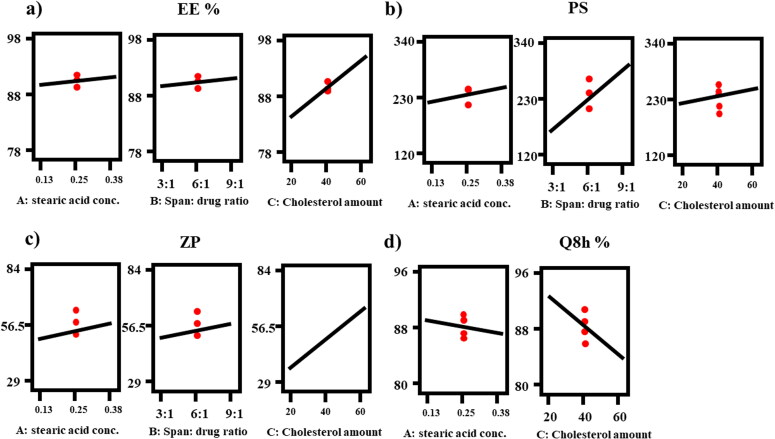
Response-plots for the effect of Factor A: stearic acid concentration (%), Factor B: span: drug ratio and Factor C: cholesterol amount (mg) on (a) EE%, (b) PS, (c) ZP and (d) Q8h (%).

The ability of the formulated novasomes to entrap considerable quantity of FTN is crucial for its potential use as an ocular delivery system (Abdelbary et al., 2016). EE % varied between (78.47 ± 0.98 and 97.92 ± 0.79%), as presented in Table 2. ANOVA analysis revealed that factor A (stearic acid concentration (%)), factor B (span 80: drug ratio) and factor C (cholesterol amount (mg)) had a positive significant effect (*p* < 0.05). The effects of all factors are graphically explained in Figure 1(a). The resulting equation in terms of coded factors was as follows:

$$\text{EE }\%=\text{ 9}0.\text{56 }+0.\text{74A }+\text{ 1}.\text{19B }+\text{ 5}.\text{58 C }–0.{\text{66 C}}^{\text{2}}$$

Considering factor A (*p* = 0.0001), stearic acid is a saturated free fatty acid (FAA) with wide application because of its safety (Singh et al., 2020). Its positive significant effect could be explained on the basis of increasing the hydrophobicity of the medium which is favorable to the hydrophobic FTN (Ahmad et al., 2019). Hydrophobicity of stearic acid arises from its long alkyl chain (C18) along with its saturation reflecting in high phase transition temperature (Tc = 69 °C), leading to the formation of less leaky vesicles resulting in higher EE%. Regarding factor B (*p* < 0.0001), increasing ratio or concentration of span 80 having low HLB (4.3) (Ruckmani & Sankar, 2010) due to its long alkyl chain of oleate moiety(C18) (Abdelbary et al., 2017), resulting in less hydrophilic holes and reducing the fluidity of the membrane by reducing the bilayers amphiphilic property (Abdelbari et al., 2021). Moreover, it was found that the EE% value increases upon increasing surfactant concentration which could be attributed to the added emulsification and stabilization impact of the lipid material in the presence of high surfactant concentration (Abdelbary & Fahmy, 2009). Finally, increasing the concentration of surfactant resulted in increasing the amount of the formed novasomes and subsequently the volume of the hydrophobic bilayer that acts as housing domain of the hydrophobic drug FTN (El-Laithy et al., 2011). Regarding Factor C (*p* < 0.0001), as the concentration of cholesterol increases, the permeability of the bilayers membrane decreases with increasing membrane rigidity resulting in high entrapment of the lipophilic drug within the bilayers (Abdelbary et al., 2017; Emad Eldeeb et al., 2019).

#### 3.1.2. Model analysis of PS

Nano-sized drug delivery systems could enhance corneal permeation and extend their efficacy (Dai et al., 2013). PS varied from (124.35 ± 0.92 to 338.95 ± 25.95 nm) as presented in Table 2. ANOVA analysis showed that factor A (stearic acid concentration (%)), factor B (span 80: drug ratio) and factor C (cholesterol amount (mg)) had a positive significant effect (*p* < 0.05). The effects of all factors are graphically explained in Figure 1(b). The resulting equation in terms of coded factors was as follows:

PS = 237.68 + 16.62A + 66.92B + 12.79C

Considering factor A (*p* = 0.0138), stearic acid is a FAA with high melting point (69 °C) which leads to greater melting viscosity and subsequently reducing the efficacy of sonication in decreasing PS. Also, there is a direct association between the PS and the formerly stated increase in the EE %, since entrapping large quantities of FTN results in increasing the distance between the bilayers, consequently PS increases (Ahmed et al., 2020; Hathout et al., 2007). Therefore increasing the concentration of stearic acid, span 80 (factor B, *p* < 0.0001) or cholesterol (factor C, *p* = 0.0493) will lead to more entrapment of the hydrophobic FTN within the bilayers as previously discussed in model analysis of EE % resulting in increasing the PS (Mosallam et al., 2021). The effect of factor C might be also explained in terms of the amphiphilic nature of cholesterol. The cholesterol intercalates inside the bilayer by placing the polar head toward the hydrophilic surface and the aliphatic chain parallel to the surfactant alkyl chains, accordingly PS increased (Emad Eldeeb et al., 2019). Also, the presence of a large amount of vesicles-forming materials relative to the hydration medium would lead to accumulation of multiple layers over each other and thus PS increased (Abdelbari et al., 2021).

#### 3.1.3. Model analysis of PDI

With regard to PDI, a value of 0 indicates homogenous system, while a value of 1 indicates highly heterogeneous system (Ahmed et al., 2020; Abdelbary & Aburahma, 2015). PDI of all the prepared formulae fluctuated between (0.06 ± 0.01 to 0.56 ± 0.02) as presented in Table 2. These results confirmed the proper uniformity of the prepared FTN-loaded formulae. ANOVA analysis revealed that all factors had a non-significant effect (*p* > 0.05) on PDI. So it was excluded from optimization criteria.

#### 3.1.4. Model analysis of ZP

Zeta potential (ZP) gives an indication about the stability of the nano-system as it represents the overall charges obtained by colloidal dispersion. As a general rule, the system with ZP around ± 30 is considered stable due to the presence of electric repulsion between particles (Muller et al., 2001). Results of Zeta potential values ranged from (-29.45 ± 1.91 to −83.30 ± 0.57) as shown in Table 2, clarifying that all formulae had enough charge to avoid accumulation of particles. ANOVA analysis showed that factor A (stearic acid concentration (%)), factor B (span 80: drug ratio) and factor C (cholesterol amount (mg)) had a positive significant (*p* < 0.05) effect on ZP. The effects of all factors are graphically explained in Figure 1(c). The resulting equation in terms of coded factors was as follows:

ZP = 54.04 + 3.78A +3.05B + 14.00C

It was detected that the absolute ZP value increases upon increasing amount of stearic acid, surfactant or cholesterol. Stearic acid (factor A, *p* = 0.0017) has free carboxylic acid group (C_17_H_35_COOH). However, span 80 (factor B, *p* = 0.0076) and cholesterol (factor C, *p* < 0.0001) have free hydroxyl groups in their chemical structure (C_24_H_44_O_6_ and C_27_H_46_O respectively). Ionization of these groups would increase the negative charge of the resulted formulae, accordingly all factors had a positive significant effect on ZP (Emad Eldeeb et al., 2019; Zubairu et al., 2015).

#### 3.1.5. Model analysis of in vitro release (Q8h)

**Figure 2.**
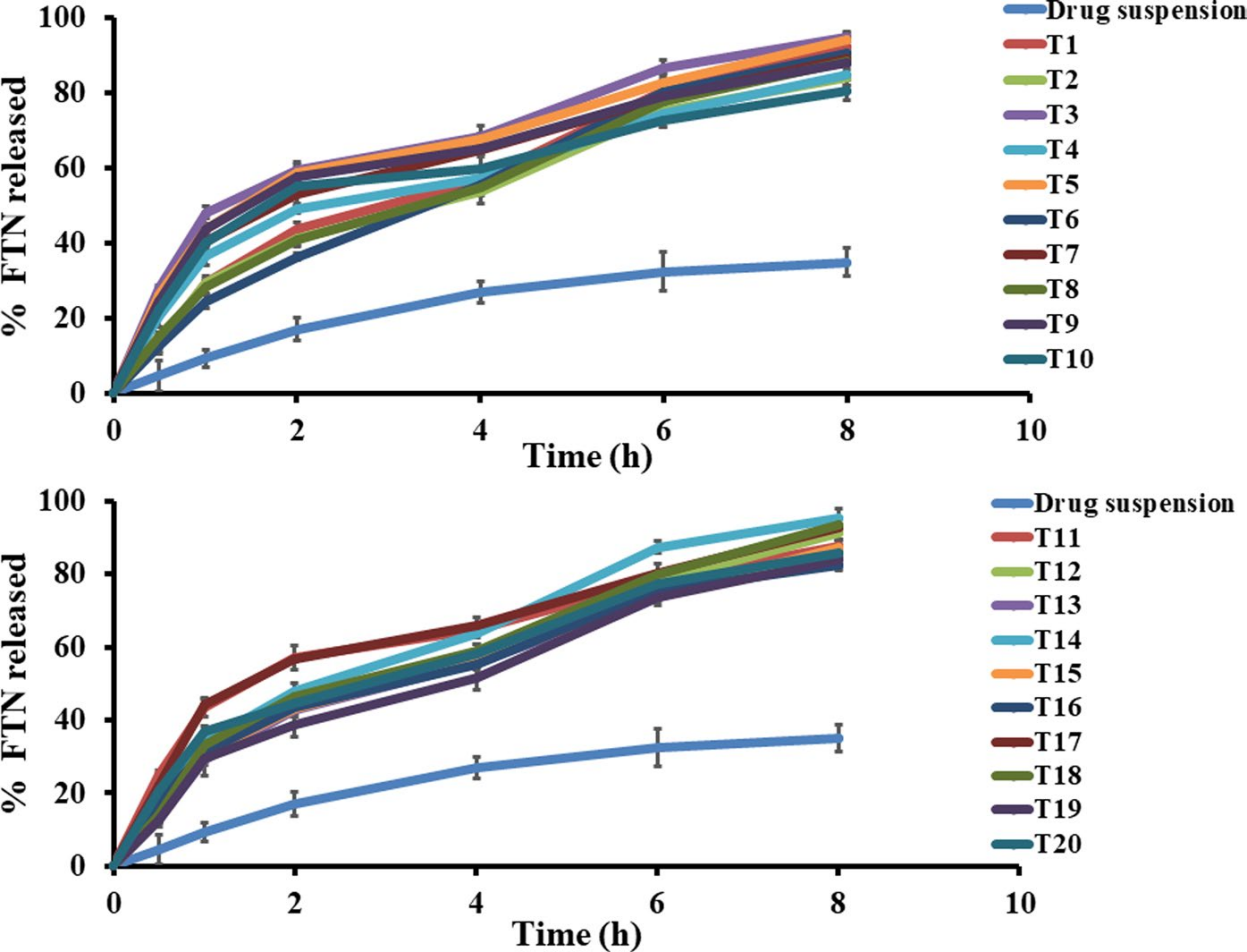
*In vitro* Fenticonazole Nitrate (FTN) release profiles of the prepared FTN-loaded formulae compared to FTN suspension at 37 ± 0.5 0 C, mean ± SD, *n* = 3.

*In vitro* release profiles of all the prepared formulae were accomplished against FTN suspension as control. Q8h of all the prepared formulae fluctuated between (80.78 ± 2.83 to 95.57 ± 2.48) as presented in Table 2. Figure 2 clarifies that the *in vitro* release profiles of all the prepared FTN-loaded formulae showed significantly faster release profile (*p <* 0.05) more than FTN suspension. This behavior was mainly attributed to the smaller particle size of the developed novasomes compared with the size of the pure drug suspension being coarse dispersion. Regarding ANOVA analysis, factor A (stearic acid concentration (%)) and factor C (cholesterol amount (mg)) had a negative significant effect on Q8h (*p* < 0.05), while factor B (span 80: drug ratio) had non-significant effect at Q8h (*p* > 0.05). The outcomes of these factors are shown in Figure 1(d). The resulting equation in terms of coded factors was as follows:

Q8h = 88.78 –1.19A – 4.77C

Both factor A (*p* = 0.0031) and factor C (*p* < 0.0001) lead to increase the hydrophobicity of the constructed formula compared to the release medium, so FTN would favor to stay inside the novasomal system. Generally, the more entrapped drug the larger the vesicle size therefore the slower the release (Ahmed et al., 2020; Abdelbary & Aburahma, 2015). Also, incorporation of stearic acid and cholesterol would decrease the formation of transient hydrophilic holes and reduce the fluidity of the lipid bilayer (Abdelbary et al., 2017). It is critical to justify the biphasic release profile of the novasomal system as the early burst phase might be due to the desorption of the unentrapped FTN that presents between the large hydrocarbon chains in the lipid bilayer of novasomal system. After that, a slower phase due to the reduced fluidity of the novasomal system by stearic acid and cholesterol (Ahmed et al., 2021). Higuchi-diffusion model is the best model to justify the release profiles (highest r-square).

### 3.2. Testing the validity of optimization process

Numerical optimization showed that the composition of the optimum formula was (stearic acid concentration = 0.37%, span 80: drug ratio = 3:1 and cholesterol amount = 60 mg with desirability =0.738). The optimum formula revealed the highest EE % (94.31 ± 2.50%), and ZP (-66.95 ± 0.92 mV) (as absolute value), Q8h > 80% (85.33 ± 1.35%) and lowest PS (197.05 ± 9.97) respectively. The validity of the optimization process was guaranteed by matching the results of the observed and predicated responses regarding EE %, PS, ZP and % drug released (Q8h) as presented in Table 4. The small % deviation as absolute value verifies our models.

**Table 4.** The optimum formula characterization.

**Response**	**Y1**	**Y2**	**Y4**	**Y5**
	**EE %**	**PS (nm)**	**ZP (mV)**	**Q8h (%)**
Observed value	94.31	197.05	−66.95	85.33
Predicated value	95.02	200.16	−68.76	82.82
% Deviation (absolute)	0.75	1.55	2.63	3.03

**Abbreviations:** EE %, percent entrapment efficiency; PS, particle size; Q8h, percent drug released after 8 hours; ZP, zeta potential.

### 3.3. In vitro characterization of the optimum formula

#### 3.3.1. Differential scanning calorimetry (DSC)

**Figure 3.**
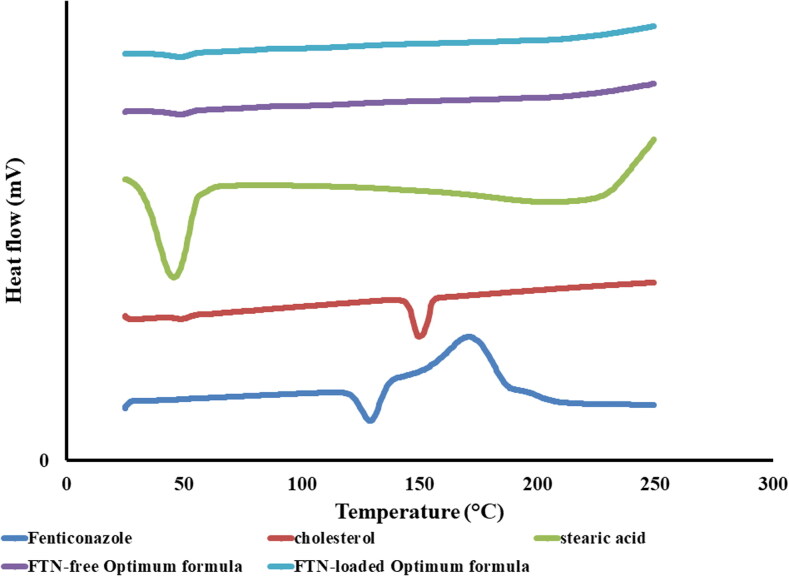
DSC thermograms of pure Fenticonazole (FTN), stearic acid, cholesterol, FTN-loaded optimum formula and FTN-free optimum formula.

DSC thermograms of FTN, stearic acid, cholesterol, lyophilized FTN-loaded optimum formula and lyophilized FTN-free optimum formula are presented in Figure 3. FTN revealed an endothermic peak at 135 °C that is related to its melting point (Kim et al., 1989; Albash et al., 2020). Stearic acid thermogram showed an endothermic peak at 55 °C (HariPrasad D et al., 2020). Cholesterol thermogram showed an endothermic peak at 38 °C and 149 °C related to its solid phase transition and melting point respectively (Jin et al., 2013). It is obvious that the disappearance of the characteristic melting peak of FTN certifies the total entrapment of FTN inside the novasomes (Shamma et al., 2019) and that FTN exists in its amorphous state (Albash et al., 2019).

#### 3.3.2. Fourier transforms infrared spectroscopy (FTIR)

**Figure 4.**
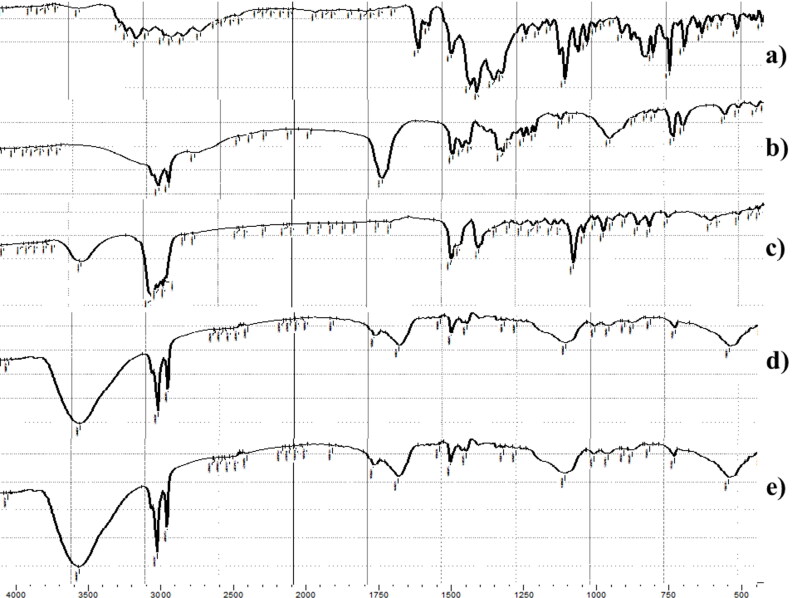
FTIR spectra of: (a) pure Fenticonazole (FTN), (b) stearic acid, (c) cholesterol, (d) FTNloaded optimum formula and (e) FTN-free optimum formula.

Figure 4 shows the FTIR spectra of FTN, stearic acid, cholesterol, lyophilized FTN-loaded optimum and lyophilized FTN-free optimum formula. FTN has a certain structure similarity with chlorinated imidazole compounds (Castro et al., 2016). The FTIR spectra of FTN are shown at 1581.63 cm^−1^ corresponding to C = N stretching, other peaks appeared at 1469.76 cm^−1^ (C = C aromatic stretch), 1091.71 cm^−1^ (C-O-C ether stretch) and 794.67 cm^−1^ (C-Cl stretch). The characteristic peaks of stearic acid are revealed at 2916.37, 2850.79 cm^−1^ (-CH_2_-) and 1701.22 (-COOH) (Zhu et al., 2016). The specific peaks of cholesterol are found at 3417.86 (-OH), in addition to bands between 2800–3000 cm^−1^ due to asymmetric and symmetric stretching of CH_2_ and CH_3_ groups respectively (Gupta et al., 2014). Disappearance of FTN characteristic peaks from the spectra of FTN-loaded optimum formula signifies the complete entrapment of the FTN inside the formula confirmed by the outcomes of DSC (Ahmed et al., 2020; Sayed et al., 2018).

#### 3.3.3. TEM microscopy

**Figure 5.**
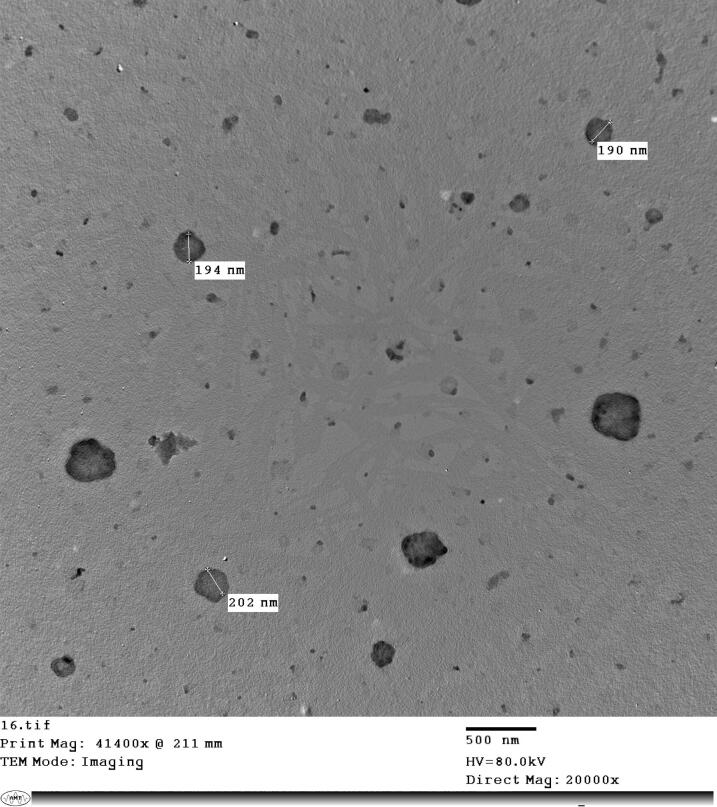
TEM image of FTN-loaded optimum formula.

Morphological evaluation of the optimum formula is presented in Figure 5. TEM provides a verification of the outcomes of Malvern particle size analyzer. PS from optimum formula measured in triplicates was (197.05 ± 9.97). TEM proved that particles were almost spherical, non-aggregated with a smooth surface and narrow size.

#### 3.3.4. pH measurement

pH of the optimum formula was 7.29 ± 0.05, ensuring the appropriateness of its ophthalmic use (pH of tears = 7.4) (Sayed et al., 2021; Fahmy et al., 2021). pH of the optimum formula also augments its efficacy since pH from 4 to 8 would considerably improve ocular permeation (Mohanty et al., 2013).

#### 3.3.5. Effect of short-term storage

At the end of the experiment, the stored vesicles did not show any clumps or changes in their physical appearance. Results of EE %, PS and ZP for fresh and stored optimum formula (3 months) are shown in Table 5. Non-significant differences (*p* > 0.05) were found in EE %, PS, ZP. The similarity factor (ƒ_2_) was equal to 87.15, indicating similar *in vitro* release profiles (Diaz et al., 2016). These results emphasizes the high stability nature of the optimum formula which could be as a result of its high negative charge (-66.95 ± 0.92 mV) that will prevent any aggregation or agglomeration of the stored vesicles (Harisa & Badran, 2015). In addition to, the small PS of the optimum formula would result in large surface area for the ionizable groups to expose their charge at the surface of the formed novasomes.

**Table 5.** Effect of short- term stability and gamma sterilization on the optimum FTN-loaded formula.

Parameter	Fresh	Storage for 3 months at 4-8 ^º^C		After gamma sterilization	
		Value	Probability (*p*)*	Value	Probability (*p*)**
**EE %**	94.31 ± 2.75	90.56 ± 3.14	0.332	93.33 ± 2.16	0.732
**PS**	197.05 ± 9.97	190.10 ± 1.13	0.431	202.80 ± 10.89	0.637
**ZP**	−66.95 ± 0.92	−63.90 ± 2.40	0.236	−66.15 ± 1.48	0.584

**Abbreviations:** EE %, percent entrapment efficiency; PS, particle size; ZP, zeta potential.

* One-way ANOVA analysis to compare between the freshly prepared and the stored optimum formula.

** One-way ANOVA analysis to compare between the freshly prepared and gamma sterilized optimum formula.

#### 3.3.6. Effect of gamma sterilization

Sterilization of ophthalmic dosage forms is essential to prevent co-infecting the patients with dangerous microbes and is less risky than aseptic processing (Sayed et al., 2021; Younes et al., 2018). No observable change in the physical appearance after gamma sterilization was detected. Moreover, EE %, PS and ZP values did not alter significantly when matched to the unsterilized control (*p* > 0.05), as presented in Table 5. The similarity factor (ƒ_2_) was equal to 77.94, indicating similar *in vitro* release profiles (Diaz et al., 2016). These results indicate that gamma sterilization will not harm the optimum formula and can be used safely as sterilization technique.

#### 3.3.7. Minimum inhibitory concentration (MIC) determination

The antifungal activity of the optimum formula and FTN suspension were evaluated *in-vitro* using *Candida albicans* ATCC 60193 as the tested organism (Albash et al., 2021). MIC for the optimum formula was found to be equal to 62.5 ug/mL while that for FTN suspension was higher than 500 ug/mL. The optimum formula showed more than 3 times higher antifungal activity than that of FTN suspension which might be due to the lower particle size and higher zeta potential values. Small PS would result in longer residence time and enhanced drug permeability. ZP affects the interaction of the formed system with the negatively charged microbial cell surface. Patil *et al*., proposed the existence of few cationic sites for nonspecific adsorption of the negatively charged particles in the form of clusters as a result of their repulsive interactions with the large negatively charged domains of the cell surface. Moreover, the adsorbed particles create a lowered charge density that may support adsorption of other free particles. Thus, the enhanced cellular uptake of negatively charged nanoparticles resulted from the nonspecific adsorption and formation of nanoparticles clusters (Patil et al., 2007). These results conform with previous studies where the PS and ZP significantly influenced the antifungal activity of the tested drugs (Ing et al., 2012). Finally, improved permeation of the optimum formula will exert higher antifungal activity leading to a lower MIC compared to FTN suspension.

### 3.4. Ex vivo characterization of the optimum FTN-loaded formula

#### 3.4.1. *Ex vivo* corneal permeation

**Figure 6.**
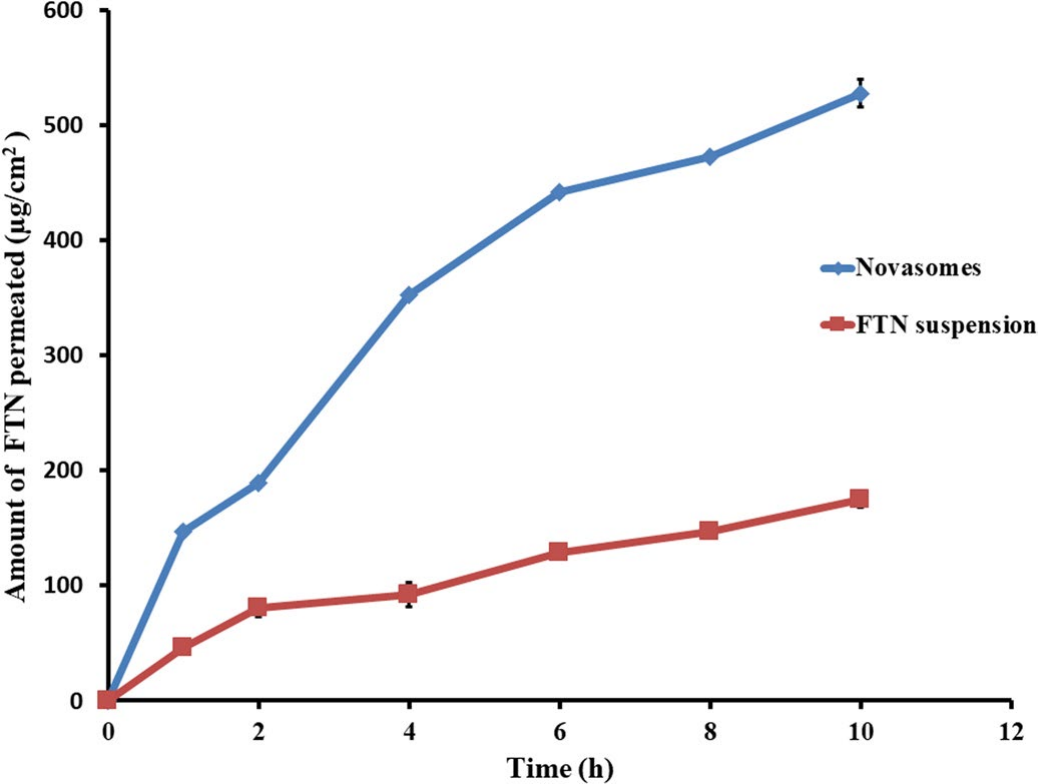
*Ex-vivo* permeation profiles of FTN-loaded optimum formula compared to FTN suspension at 37 ± 0.5 0 C, mean ± SD, *n* = 3.

The permeation profiles of the optimum and FTN suspension are shown in Figure 6. The optimum formula displayed improved quantity of FTN permeated after 10 h (Q_10h_-_permeation_ of the optimum formula = 527.98 ± 12.00 µg/cm^2^) compared to (Q_10h_-_permeation_ of FTN suspension = 174.66 ± 6.94 µg/cm^2^). The optimum formula showed superior flux (J_max_ of the optimum formula = 52.80 ± 1.20 µg/cm^2^/h) compared to (J_max_ of FTN suspension = 17.47 ± 0.69 µg/cm^2^/h). One-way ANOVA showed that the optimum formula had significantly (*p* < 0.05) higher amount permeated after 10 h and flux compared to FTN suspension. The enhancement ratio of the optimum formula was (3.02). These results emphasize higher permeation of FTN from the optimum formula. The superior permeation results of the prepared novasomes might be due its small PS which increase the residence time and facilitates the passage through the hydrated network of the corneal stroma (Younes et al., 2018; Mosallam et al., 2021). The presence of stearic acid also contributes to better permeation since saturated fatty acids with high melting point had an improved permeation rate across the biological membranes. FFA can incorporate rapidly into the lipid membrane and increase the curvature stress that results in formation of lipid instabilities and enhancing its permeability (Singh et al., 2020). Moreover, cholesterol acts as penetration enhancer that facilitates the diffusion of the formed novasomes (Emad Eldeeb et al., 2019). Finally, surfactants could improve the permeation by loosening the tight junctions of the corneal epithelium (Younes et al., 2018).

#### 3.4.2. *Ex vivo* corneal hydration level

The corneal hydration level (HL%) was calculated to estimate the injury initiated to the corneal tissues after the *ex vivo* permeation study. HL% of the normal healthy cornea is between (76–80%) (Huang et al., 2017). HL% of the optimum formula and FTN suspension were found to be equal to (78.50 ± 0.37 and 79.25 ± 0.48 respectively). One-way ANOVA test revealed absence of significant difference (*p* > 0.05) in % HL obtained from the optimum formula and FTN suspension. Consequently, the optimum formula could be counted safe and non-harmful to the eye (Moustafa et al., 2017).

### 3.5. In vivo characterization of the optimum FTN-loaded formula

#### 3.5.1. Ocular irritancy test

Results of this test showed that the optimum formula did not display any sign of redness, inflammation or increased tear production for 24 h. Consequently, the optimum FTN-loaded novasomes could be considered tolerable and non- irritant to the eye (Abdelbary et al., 2017).

#### 3.5.2. In vivo corneal tolerance

**Figure 7.**
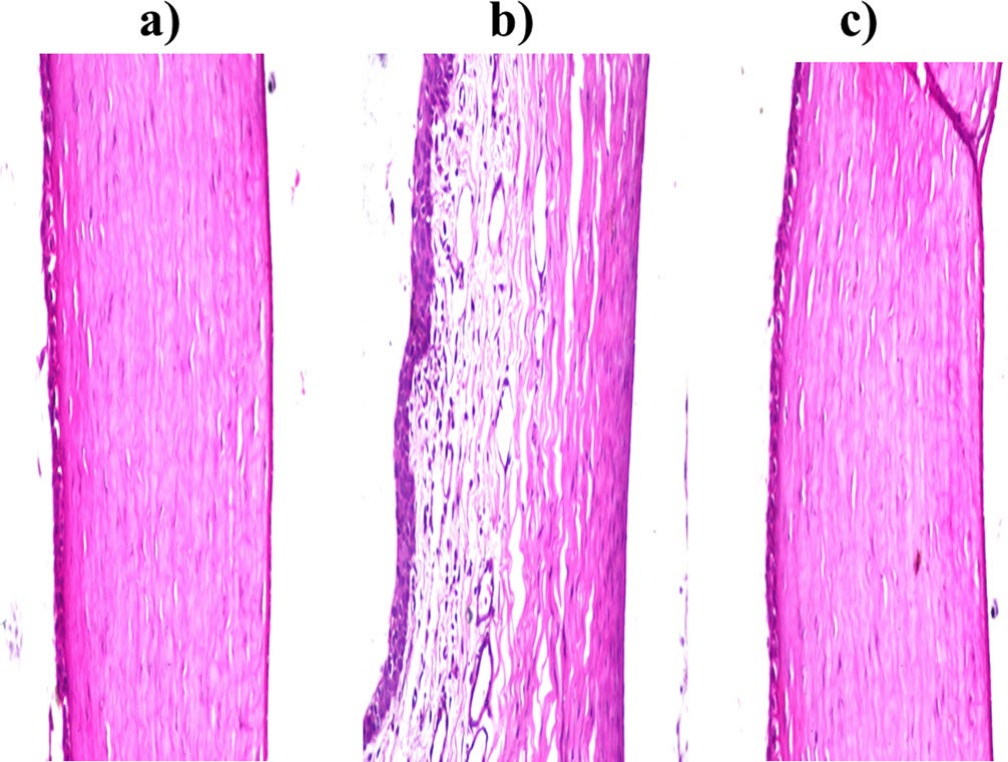
Photomicrographs of the rabbits’ corneas after instillation of; a) Normal saline solution (negative control), b) Isopropyl alcohol (positive control) and c) FTN-loaded optimum formula.

Considering corneal tissues exposed to normal saline as negative control; no histopathological change with normal histological structure of the covering lining epithelial cell layer, the underlying stroma, and the last layer of the endothelium, as shown in Figure 7(a). Regarding corneal tissues exposed to isopropyl alcohol as positive control; deformed and separated epithelial layers were detected, as shown in Figure 7(b). Considering corneal tissues exposed to the optimum formula; no histopathological modification in the cornea, iris, retina, or sclera, as shown in Figure 7(c). As a conclusion, the optimum FTN-loaded novasomes are considered safe be applied to the eye without any injuring or inflammation consequences (Sayed et al., 2020).

#### 3.5.3. In vivo corneal uptake

**Figure 8.**
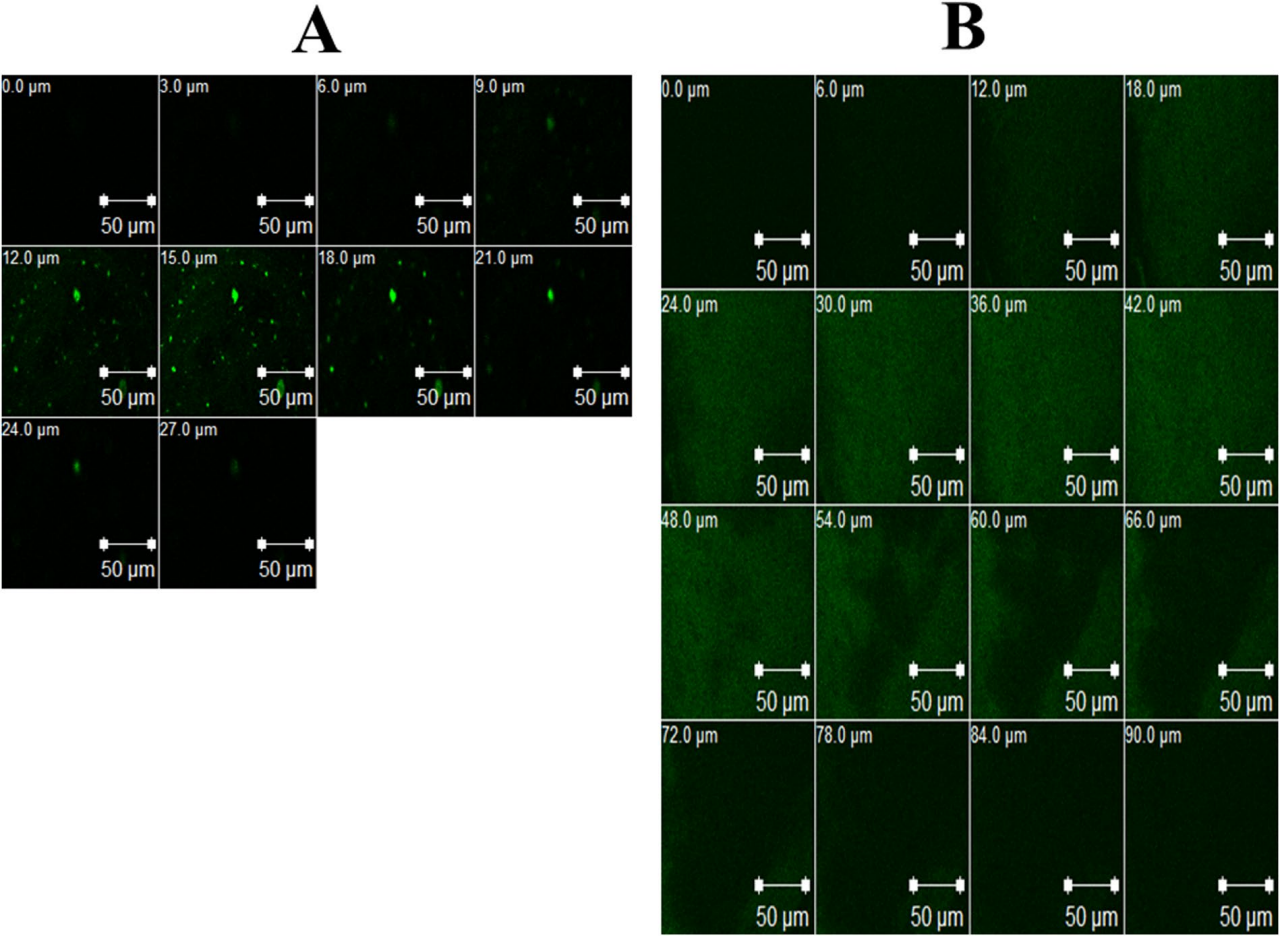
Confocal laser scanning micrographs (CLSM) of rabbits’ corneas after instillation of: a) RhB aqueous solution and b) RhB-loaded optimum formula.

To study the ability of the optimum formula to enhance the corneal permeation of FTN, CLSM was employed to detect the transcorneal behavior of RhB-loaded formulae after instillation, by tracing fluorescence signals inside the corneal tissues. Upon examining CLSM micrographs, it is clear that the RhB-loaded optimum formula demonstrated greater penetration (90 μm) than RhB-loaded aqueous solution (27 μm), as shown in Figure 8. This fits with the findings of earlier *ex vivo* permeation study. Carrying the antifungal drug (FTN) into the stromal layer providing a good treatment option for deep fungal infections (Younes et al., 2018).

#### 3.5.4. Susceptibility test

**Figure 9.**
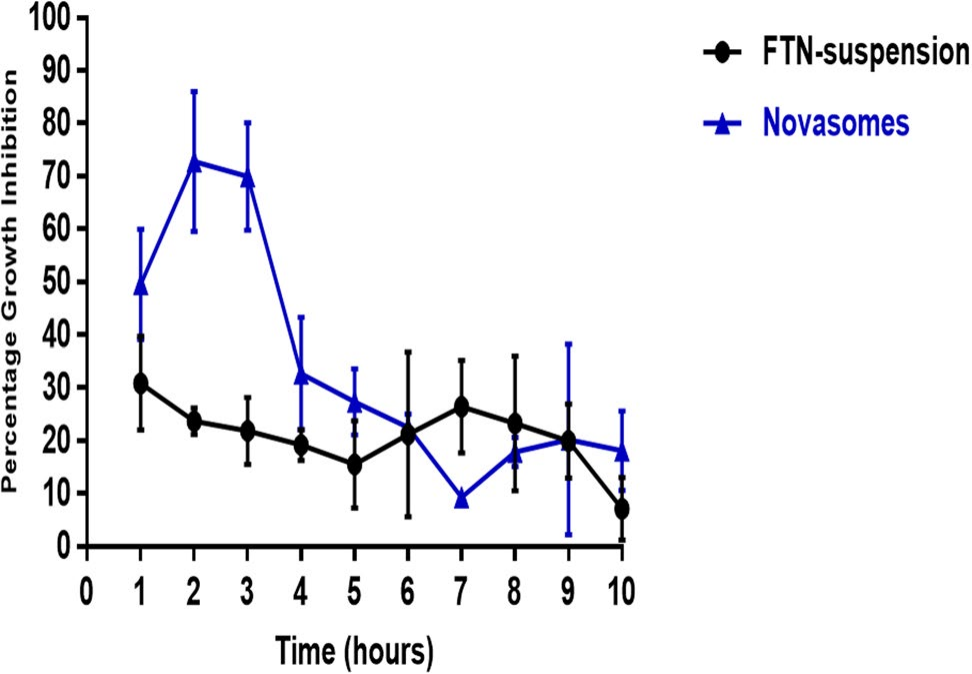
Percentage inhibition of Candida albicans growth produced by FTN-loaded optimum formula compared to FTN suspension in rabbit external ocular tissue.

The optimum formula and FTN suspension were evaluated *in vivo* using *Candida albicans* ATCC 60193 as the test organism. Percentage of growth inhibition of *Candida albicans* was related to the drug’s retention time on the eye surface following the administration, as shown in Figure 9. Growth inhibition percentage of the optimum formula reached the maximum (72.77 ± 13%) two-hours post-administration and then decreased gradually. On the other hand, the FTN suspension reached a maximum of 30.83 ± 8.8% one-hour post-administration and then showed almost constant level from the second hour until the 9^th^ hour of the study period (27 ± 2.5% − 20.16 ± 7.02% from 2 h − 9 h). The growth inhibition percentage of the optimum formula was significantly higher than that of FTN suspension until the fifth hour post-application (Student’s *t*-test, *p* < 0.05, *p-*value =0.019). The optimum formula significantly sustained the antifungal activity of FTN on the ocular surface for a relatively longer time with an area under the curve of about 1.6 fold higher than that of FTN suspension (AUC1h-10h = 306.5 and 190.2, respectively).

## 4. Conclusions

In this research, FTN-loaded novasomes were fruitfully prepared via ethanol injection technique. The optimum formula proved minute particle size (197.05 ± 9.97 nm) with spherical morphology, great percent entrapment efficiency (94.31 ± 2.50%), accepted zeta potential (-66.95 ± 0.92 mV), high % *in vitro* release (Q8h) (85.33 ± 1.35%) and high physicochemical stability. The complete entrapment of FTN inside the optimum formula was ensured by DSC and FTIR studies. Suitability of the optimum formula was proved through pH measurement, *ex vivo* corneal hydration level, enhanced *ex vivo* corneal permeation (Q_10h_-_permeation_ = 527.98 ± 12.00 µg/cm^2^) and high stability after gamma irradiation. Efficacy of the optimum formula was proved through *in vitro* MIC determination. Further *in vivo* tests including; ocular irritancy test, *in vivo* corneal tolerance test, *in vivo* corneal uptake and susceptibility test revealed the validity of FTN-loaded novasomes as a favorable method to enhance FTN ocular delivery and thereby its antifungal activity.
